# Legal Challenges in Precision Medicine: What Duties Arising From Genetic and Genomic Testing Does a Physician Owe to Patients?

**DOI:** 10.3389/fmed.2021.663014

**Published:** 2021-07-26

**Authors:** Scott P. McGrath, Arthur E. Peabody, Derek Walton, Nephi Walton

**Affiliations:** ^1^CITRIS and the Banatao Institute, University of California, Berkeley, Berkeley, CA, United States; ^2^Hooper, Lundy & Bookman, Professional Corporation, Washington, DC, United States; ^3^Walton Legal Professional Limited Liability Company, Salt Lake City, UT, United States; ^4^Intermountain Healthcare, Salt Lake City, UT, United States

**Keywords:** precision medicine, scope of practice, genetic testing, genomics, physicians

## Abstract

Precision medicine is increasingly incorporated into clinical practice *via* three primary data conduits: environmental, lifestyle, and genetic data. In this manuscript we take a closer look at the genetic tier of precision medicine. The volume and variety of data provides a more robust picture of health for individual patients and patient populations. However, this increased data may also have an adverse effect by muddling our understanding without the proper pedagogical tools. Patient genomic data can be challenging to work with. Physicians may encounter genetic results which are not fully understood. Genetic tests may also lead to the quandary of linking patients with diseases or disorders where there are no known treatments. Thus, physicians face a unique challenge of establishing the proper scope of their duty to patients when dealing with genomic data. Some of those scope of practice boundaries have been established as a result of litigation, while others remain an open question. In this paper, we map out some of the legal challenges facing the genomic component of precision medicine, both established and some questions requiring additional guidance. If physicians begin to perceive genomic data as falling short in overall benefit to their patients, it may detrimentally impact precision medicine as a whole. Helping to develop guidance for physicians working with patient genomic data can help avoid this fate of faltering confidence.

## Introduction

As precision medicine proliferates in the provision of medical care (1, 2), a critical component is the use of an individual's genomic information in diagnosis and individualized treatments (3–5). Yet physicians face unique challenges. Physician knowledge of genetics largely remains incomplete (6) and there is a gap in their ability to interpret genetic results in comparison to genetic specialists (7). Genomics is rapidly changing, both at the focused level of genetic tests in the wider scope of genomic tests, making it difficult to keep abreast of the latest developments (8, 9). Studies have shown that labs can yield both inconsistent results and more information than the typical physician can reasonably digest (10–12). Clinical applications can be confusing and not readily available in many cases. Polygenic risk scores, e.g., may be adding complexity to the corpus of knowledge a health provider must be aware of, and also may risk exacerbating health disparities by catering certain treatments to different racial/ethnic groups (13, 14). There are few physicians that possess the education, skills, and experience to take full advantage of genetic testing, including what test is optimal to select (15–18). Numerous ethical and privacy concerns lurk over the shoulder of every physician choosing to use genetic information (19–23). This article seeks to analyze the current state of physician liability of using genetic information. In the field of genetic testing, there are more questions than answers. In many areas, there is a need to analyze the specific facts at issue and develop carefully crafted solutions to avoid the ever-looming specter of legal liability. There is currently a lack of case law in the United States, Europe, or in the United Kingdom that directly addresses these issues (24–26), therefore, we focus on legal principles established in American jurisprudence and those few reported cases in the courts of the United States to provide a framework to explore this topic.

## Legal Standards

The legal liability of physicians is generally governed by the common law of negligence. In general, a physician is negligent or liable for challenged conduct when they fail to exercise reasonable judgment and departs from generally acceptable standards of practice. In other words, a physician is negligent when he or she fails to follow generally accepted practice in any particular medical domain. State law is especially important because state courts set the parameters of what kind of claims can be sought and on what basis. Some genetic legal cases have required explicit statutes (27), while others have relied on common law notions of negligence or personal injury law absent any statute (28). Yet, in precision medicine, the question may be far more complicated. When the interpretation of genetic information is at issue, there may be no generally accepted practice or standard. Physicians are constantly challenged by what to do with genetic knowledge that is available but may not be fully understood or for diseases for which there is no known treatment.

At what specific point does clinical genetic knowledge become a standard of practice? This issue is being tested in both federal and state courts. In *Williams v. Quest/Athena* (29), the plaintiff sued the laboratory that conducted genetic testing of the plaintiff's son, who subsequently died. The plaintiff has argued that two genetic studies conducted prior to the lab's allegedly erroneous reporting that the variant was of “unknown significance.” This misdiagnosed genetic mutation led directly to the child's inappropriate treatment and death. Are two studies sufficient to establish a standard of practice? Do two studies establish that the variant in question is “significant” and must be properly identified by the lab and addressed by the physician? The federal court ultimately found for the Defendants holding, among other things, that there was insufficient evidence that the plaintiff's son's variant was pathogenic and, as a result, no reasonable jury could find negligence (30). This case points out how unclear the standard for legal liability can be under the current state of the law.

Some have suggested that evidence-based medicine should determine when there is sufficient evidence to find a variant to be pathogenic and, as a result, provide direction to physicians navigating unfamiliar genetic or genomic results. However, consensus acquired through evidence-based medicine relating to genomic data is still maturing. As a result, this approach does not always provide medical practitioners guidance on what steps to take. Nonetheless, efforts are being made to determine if the principles of evidence-based medicine can be effectively applied to genomic data (31) and how exactly genomics should be integrated into healthcare (32–34).

## Physician Challenges

Yet the challenges do not end with the question of a legal standard. How does a physician keep up or remain current with the evolving body of genetic knowledge? There are five categorizations of genetic variant: pathogenetic, likely pathogenic, variant of uncertain significance (VUS), likely benign, and benign. The usage of “likely” is defined as “a >90% certainty that a specific variant is pathogenic or benign” (35). A 25-gene cancer susceptibility panel will report at least one VUS about 33% of the time (36). These VUS can obfuscate a physician's duty and generate several questions.

- Are physicians responsible for tracking reported VUS, in case they become classified as “significant” for both current and previous patients?
- Does the nature of the duty change when the doctor is the patient's “primary physician?”
- Do general practitioners or genetic specialists owe the same or a different duty based on their expertise in genetics?

A further confounding factor is the gap, sometimes indefinite, between the capacity to diagnose using genetic tests and the capacity to treat due to the lack of effective therapies for any number of genetically linked conditions (37, 38).

But the physicians' quandary does not stop here. As the ability to interpret genetic variations grows, the physician is faced with the fact that known variants that have the potential for disease may never lead to the disease, including patients with a genetic disease for which there is no known treatment (39). What duty does a physician owe to these patients even when it is understood that known variants may never lead to the disease in particular, individual patients?

The challenge of genetic knowledge is knowing what to do with most genetic information. For example, women who test positive for BRCA-1 or BRCA-2 pathogenic variants, may undergo prophylactic mastectomy because of their fear of developing an aggressive cancer. However, some of these patients may have been either misdiagnosed or do not fully appreciate the uncertain nature of the genetic mutations (40, 41). Some have undergone unnecessary surgery; others may have escaped breast cancer. How do we balance the benefits and potential risks of harm of genetic knowledge? This is a question society, in general, and physicians, in particular, have only begun to address.

These alleged unnecessary mastectomies have led to litigation. In *Moore v. Curry County Health* (42), the plaintiff alleged that the physician misread her genetic tests and even if the variant was interpreted correctly, the variant did not lead to breast cancer. The facts as alleged, if true, may form a plausible claim for damages as the physician may not have followed the standard of practice in interpreting the plaintiff's genetic test results, but like all cases in this field to date, there is always a wrinkle. The plaintiff had a family history of breast cancer. Would the family history of breast cancer have justified the mastectomy, notwithstanding whatever the genetic testing may have revealed? In *Moore v. Curry*, the plaintiff settled for $600 k+ in damages without disclosing the terms of the settlement (43).

## Advanced Genomic Techniques and Physician Duty

Whole exome sequencing (WES) is becoming popular for diagnosing patients with complex disease. However, tests often return results outside of the condition for which it was originally ordered (44). Does the physician have an obligation to review all of the findings even though they were not ordered and may lack relevancy to their diagnosis? Pharmacogenomic testing results can guide prescribing. However, physicians are unlikely to look at a WES test report to assess for pharmacogenomic findings prior to prescribing a medication (45). Is the physician liable for not reviewing WES data in a patient's chart, if the failure to do so leads to harm of the patient? This issue is exacerbated by the volume of information that may be provided and the numerous findings of “variant of unknown significance” that may be contained in the report. At least one court has found liability for failing to diagnose medical issues that may be disclosed during the course of tests and other measures a physician undertakes to resolve a patient's other issues. The courts have labeled the medical issues discovered in the course of other tests as “incidental findings.” For example, in *Lo v. Burke* (46), a court found a radiologist liable for not detecting a pancreatic tumor when searching for a tumor in the liver.

In addition to the aforementioned challenges, WES may not adequately cover all genetic regions (promotors or intronic areas, e.g.,) that are important to a certain pharmacogenomic result or secondary finding (47). What happens if the physician does review the pharmacogenomic data from WES but is unaware of the limitations of the regions covered and, as a result, fails to order the more comprehensive test that would have more appropriately guided the prescribing? Legal precedent has not yet addressed this and other issues relating to the extent of physician liability that may arise from the use of WES in diagnosing and planning a patient's course of treatment.

In confronting test results the physician must decide both what to disclose and who else to advise. For example, if a patient's test reveals a significant variant known to be linked to a disease that is potentially hereditary in nature, is the physician under any obligation to advise the patient's children or any other family member, e.g., the patient's siblings, of the variant, and their potential exposure to the disease? Here, the courts have reached different results.

In *Pate v. Threlkel* (48), the court ultimately held that the physician had the duty to advise the patient to warn her children of their increased risk of disease due to the genetic makeup of their mother – but no duty of the physician to advise the children directly. This holding was based on Florida's “HIPAA statute” barring the disclosure of protected health information absent consent of the patient. HIPAA protects most private health information except for certain explicit reasons, including treatment. This exception, however, applies only to the patient. HIPAA does authorize a patient to provide “informal” consent to a physician to disclose genetic information to potentially affected family members, but state law, as in Threlkel, may impose more formal requirements or bar the disclosures.

In *Safer v. Estate of Pack* (49), the court held that a physician has a duty to warn all members of a patient's immediate family of the patient's potentially genetically transferrable disease – in spite of HIPAA. The American Medical Association provides some guidance to physicians who need to counsel patients about sharing genetic test results to family members (50). The American Society of Human Genetics' statement suggests that a physician may be justified in warning family members directly if the patient declines to cooperate in circumstances posing serious risks to family members (51). The alleged duty to warn may have serious unintended consequences. For example, in diagnosing potential birth defects in an unborn child, both parents may be subjected to genetic testing. The result may call into question the paternity of the child. The question of scope of physician duty is again brought into focus when dealing with families.

- What bearing does infrequent application of genetic knowledge impact a physician's duty to warn a patient's children and family members?
- What guidance should be provided to physicians in order to make such decisions when faced with a multitude of “variants of unknown significance” results?
- When does a physician have a duty to recommend genetic testing in face of a patient's history of disease generally associated with a genetic mutation?

While questions surrounding family consultations are numerous, the duty to recommend genetic testing is not a novel issue for courts to address. Case precedent exits extending as far back as the early 1980's holding that the failure to recommend genetic testing in appropriate circumstances, e.g., a family history of a genetically linked disease, constitutes a departure from the standard of care (52). State courts have found a similar duty. In *Downs v. Trias* (53), the court found a physician liable for negligence when he failed to recommend genetic testing to a woman who died of ovarian cancer where her family had a history of cancer.

## Conclusions

In summary, physicians face many challenges in the interpretation of genetic testing results given the current state of knowledge. The issue is only heightened because the field is changing rapidly with ever-increasing information. Physicians carry enormous responsibility when they enter the arena of genetic testing. The law is only beginning to articulate the duties each must fulfill. Even though, there are legal challenges that are presented with these new capabilities. As legal challenges associated with precision medicine are assessed, it is critical to not overlook the risk associated with avoiding new technology which may incur even larger liability. A recent review of genomic malpractice cases in the United States through the end of 2016 showed that most cases were not based on misinterpretation of genetic variants but were related to failure to perform genetic testing or failure to act on the results of genetic testing. In fact, it suggested that 57% of the genomic medical malpractice cases could have been avoided if genetic testing had been performed when the patient first presented with the condition (54). There is an expanding list of more 7,000 rare diseases, a majority of which are believed to have a genetic cause (55). Many of these diseases are actionable in that they have specific treatments catered to the genetic cause; for others appropriate management is just beginning to be developed or understood. Failure to test for and treat these diseases appropriately can lead to poor outcomes for patients and significant liability for providers. It is important that new technologies are embraced for the benefits they can offer and used cautiously, for fear of these technologies must not become a barrier to providing the best possible patient care.

## Data Availability Statement

The original contributions presented in the study are included in the article/supplementary material, further inquiries can be directed to the corresponding author/s.

## Author Contributions

NW and AP performed the primary research on the cases and constructed the initial draft manuscript. DW assisted with the legal reviews and contributed to the writing about legal cases. SM contributed to both the writing and research and led the revision and preparation of the final manuscript. All authors contributed to the article and approved the submitted version.

## Conflict of Interest

AP was employed by the company Hooper, Lundy & Bookman, and DW was employed by the company Walton Legal PLLC. The remaining authors declare that the research was conducted in the absence of any commercial or financial relationships that could be construed as a potential conflict of interest.

## Publisher's Note

All claims expressed in this article are solely those of the authors and do not necessarily represent those of their affiliated organizations, or those of the publisher, the editors and the reviewers. Any product that may be evaluated in this article, or claim that may be made by its manufacturer, is not guaranteed or endorsed by the publisher.

## Abbreviations

VUS: Variant of Uncertain Significance
BRCA-1: Breast Cancer Gene 1
BRCA-2: Breast Cancer Gene 2
HIPAA: Health Insurance Portability and Accountability Act
AMA: American Medical Association
ASHG: American Society of Human Genetics
NIH: National Institutes of Health.
